# Transparent Self-Cleaning Coatings Based on Colorless Polyimide/Silica Sol Nanocomposite

**DOI:** 10.3390/polym13234100

**Published:** 2021-11-25

**Authors:** Yun-Je Choi, Ju-Hee Ko, Seung-Won Jin, Hyun-Soo An, Dam-Bi Kim, Kang-Hoon Yoon, Hyun-Woo Kim, Chan-Moon Chung

**Affiliations:** Department of Chemistry, Yonsei University, Wonju 26493, Gangwon-do, Korea; cyj5560@yonsei.ac.kr (Y.-J.C.); kojuhee0305@yonsei.ac.kr (J.-H.K.); jinsw0906@yonsei.ac.kr (S.-W.J.); hyunsoo.an@yonsei.ac.kr (H.-S.A.); dam818@yonsei.ac.kr (D.-B.K.); ykh9916@yonsei.ac.kr (K.-H.Y.); skyna123@yonsei.ac.kr (H.-W.K.)

**Keywords:** self-cleaning, transparent, hydrophobic, polyimide, silica sol, nanocomposite

## Abstract

We herein report transparent self-cleaning coatings based on polyimide-fluorinated silica sol (PIFSS) nanocomposite. Polyamic acid-silica sol (PASS) suspensions were synthesized by adding four different amounts of a silica sol suspension to each end-capped polyamic acid solution. The PASS suspensions were spin-coated on glass slides, thermally imidized and treated with triethoxy-1*H*,1*H*,2*H*,2*H*-perfluorodecylsilane (TEFDS) to prepare PIFSS coatings. The PIFSS coatings showed high resistance to separation from glass substrates and thermal stability. Furthermore, the PIFSS coatings on the glass substrate could be cleanly removed using polar aprotic solvents and repeated coating was possible. As the amount of silica sol particles in the PIFSS coating was increased, the hydrophobic contact angle increased. Among them, PIFSS-10 and PIFSS-15 coatings showed nearly superhydrophobic contact angles (144° and 148°, respectively) and good self-cleaning property. It was confirmed by SEM and AFM studies that their hydrophobic and self-cleaning properties are due to uniform particle distribution and relatively high surface roughness. PIFSS-10 coating showed a high transmittance value (88%) at 550 nm and good self-cleaning property, therefore suitable as a transparent self-cleaning coating. The advantages of the coating are that the fabrication process is simple, and the substrate is reusable. The PIFSS coating is expected to be applied in solar cell panels, windows, lenses and safety goggles.

## 1. Introduction

Coating surfaces usually get contaminated through the accumulation of dirt. Recently, extensive research has been performed to develop superhydrophobic surfaces having water contact angles higher than 150° and water roll-off angles smaller than 10° for efficient self-cleaning applications [1,2,3,4,5]. On a superhydrophobic coating, water drops easily roll down by slight tilting. The rolling water droplets wash away dirt particles from the superhydrophobic surface, which would give an advantage of cost-effective maintenance. Such superhydrophobic coating surfaces find applications not only in self-cleaning, but also in anti-fouling, anti-corrosion, anti-icing and anti-freezing [6].

If the superhydrophobic coatings are even transparent, the range of possible application could be expanded to window and door glasses, skyscrapers, goggles, windshields and solar cell panels [3,7,8]. For a representative example, a solar cell converts a part of incident sunlight into electrical energy which is a clean and renewable energy. However, solar cell efficiency can be greatly reduced due to the accumulation of dirt on solar cell panels. This is a common issue faced all over the world and various time-consuming and costly methods are employed to clean the solar cell panels regularly. Optically transparent, durable and self-cleaning superhydrophobic coatings on solar cell panels are required to improve the solar cell performance.

Micro/nano-sized surface roughness and low surface energy are known to be required for superhydrophobic surfaces. However, optical transparency and surface roughness are generally contradictory properties [4,9]. The coating surface should be sufficiently rough to achieve superhydrophobicity, while higher surface roughness can reduce its transparency. So far, the transparent superhydrophobic coatings have been developed with porous silica capsules [10], organically modified silica [11,12,13], hollow silica nanoparticles [14], nanoporous silica layer/silica nanoparticle layer [15], organically modified hollow silica nanoparticles [16], hydrophobic/hydrophilic dual-sized silica particles [9], methyltrimethoxysilane based silica [17,18], nano-sized titanium oxide and polyolefin [19], long-chain fluoroalkylsilane [6], phase-separated polydimethylsiloxane (PDMS) [20], PDMS/graphite composite [21], fluorinated polymer containing silica particles [22,23], monodisperse poly(methyl methacrylate) particles [24] and polystyrene containing fluorinated silica nanoparticles [25]. However, despite successful achievement of superhydrophobicity along with high transparency in the conventional research, there have been no reports on transparent superhydrophobic coatings that is easily removable so that the substrate can be reused. For example, in the case of solar cell panel, the reuse of the substrates (generally tempered glasses) can contribute to the environment protection and reduction of maintenance cost.

Transparent colorless polyimides are considered to be a good candidate as matrix polymer of transparent self-cleaning coatings because of good film-forming property [26,27] and good weather resistance [28,29]. In the present study, we first developed a transparent self-cleaning coating based on colorless polyimide/fluorinated silica sol (PIFSS) nanocomposite. Compared to the conventional transparent self-cleaning coating, our coating system have an advantage of facile removal of the coating after use, which can make the glass substrate reusable. Another advantage of our system is that coating process is relatively simple without any mold or costly instrument. Herein PIFSS nanocomposite coatings were prepared and characterized for transparency, adhesion to substrate, pencil hardness, surface morphology and surface roughness. Preliminary evaluation of self-cleaning ability was also conducted.

## 2. Materials and Methods

### 2.1. Materials

4,4′-(Hexafluoroisopropylidene)diphthalic anhydride (6FDA), tetraethyl orthosilicate (TEOS), aqueous ammonia solution (32 wt%) and triethoxy-1*H*,1*H*,2*H*,2*H*-perfluorodecylsilane (TEFDS) were purchased from Merck-Korea (Seoul, Korea) and used as received. [(3-Triethoxysilyl)propyl]succinic anhydride (3TPA) and 2,2′-bis(trifluoromethyl)benzidine (TFMB) were purchased from Tokyo Chemical Industry (Tokyo, Japan) and used as received. Dimethylacetamide (DMAc), dimethyl sulfoxide (DMSO) and technical-grade ethanol were purchased from Ducksan Pure Chemical (Seoul, Korea) and used as received.

### 2.2. Synthesis of PASS Suspensions

**Figure 1:**
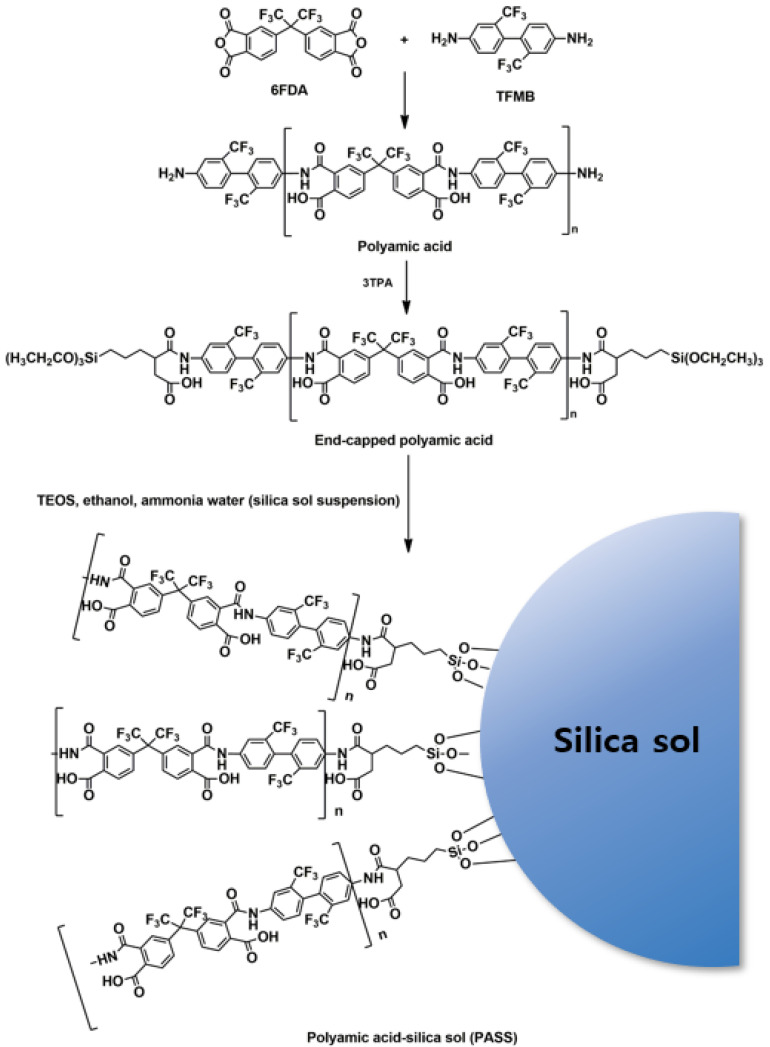
Synthesis of PASS suspensions.

The synthesis of polyamic acid-silica sol (PASS) suspensions is illustrated in Figure 1. TFMB (12.72 g) and DMAc (170 g) were added to a 250-mL one-neck round-bottom flask and were stirred at room temperature under Ar gas for 30 min. 6FDA (17.28 g) was added to the flask and the resultant solution was stirred at room temperature under Ar gas for 3 h to prepare a polyamic acid solution. The mol ratio of TFMB/6FDA was 1/0.98 and solid content was 15 wt%. After 3TPA (1.06 g) was added, the resulting solution was stirred for 1 h to synthesize 3TPA end-capped polyamic acid. To another two-neck round-bottom flask with a reflux condenser were added TEOS (2 g), ethanol (16 g), and aqueous ammonia solution (2 g). The mixture was stirred at 60 °C for 1 h to give a silica sol suspension. Different amounts (5 g, 10 g, 15 g or 20 g) of the silica sol suspension was added to each 200 g of end-capped polyamic acid solution (Table 1) and stirring was conducted at 50 °C for 3 h to obtain PASS suspensions (PASS-5, PASS-10, PASS-15 and PASS-20, respectively).

### 2.3. Preparation of PIFSS Coatings

**Figure 2:**
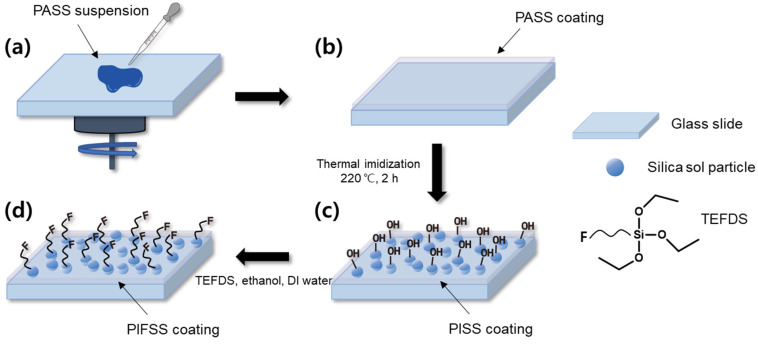
Preparation of self-cleaning PIFSS coatings: (**a**) spin-coating of a PASS suspension on a glass slide, (**b**) PASS coating on the glass slide, (**c**) PISS coating prepared by thermal imidization, and (**d**) PIFSS coating prepared by condensation reaction of hydroxyl groups with hydrolyzed TEFDS.

The preparation of polyimide-fluorinated silica sol (PIFSS) coatings is illustrated in Figure 2. The PASS suspension was coated on 2.5 × 7.5 cm^2^ glass substrates using a spin coater (ACE-200, Dong Ah Trade Corp, Seoul, Korea). The coatings were targeted to have about 1-μm thickness at 3000 rpm for 120 s. The resultant PASS coatings were heated at 220 °C for 2 h to remove residual solvent and simultaneously to imidize, affording polyimide-silica sol (PISS) coatings. The PISS coatings were placed in a vacuum oven containing a mixture of TEFDS, ethanol and deionized water in a container, and heated at 200 °C for 3 h to obtain PIFSS coatings. The appearance of PIFSS coatings are summarized in Table 1.

### 2.4. Characterizations and Properties Evalution

The PASS and PIFSS samples were examined using Fourier transform infrared (FT-IR) spectrometer (Spectrum One B, PerkinElmer Co., Waltham, MA, USA). Water contact angles of PIFSS coatings were measured on a drop shape analyzer (DSA25, KRUSS, Hamburg, Germany). UV/Vis spectrometer (Lambda 25, PerkinElmer Co., Waltham, MA, USA) was used to determine optical transmittance of PIFSS coatings. The surface images and surface roughness of PIFSS coatings were obtained by field-emission scanning electron microscope (FE-SEM 7800F, JEOL Ltd., Tokyo, Japan) and atomic force microscope (Park NX10, Park Systems Corp., Suwon, Korea). Thermogravimetric analysis (TGA) was carried out on a TGA-55 (TA instruments, New Castle, DE, USA) under a nitrogen flow of 50 mL/min at a heating rate of 10 °C/min. Pencil hardness was measured using an electric pencil hardness tester (CT-PC2, Coretech, Uiwang, Korea).

### 2.5. Self-Cleaning, Adhesion Cross-Cut and Pencil Tests

A self-cleaning test for PIFSS coatings on glass slides (7.2 × 2.5 cm^2^) was conducted. The coating samples were tilted at an angle of 20°. A standard sand (0.2 g) specified in ISO 679 was sprayed on them, and then 0.7 mL of DI water was dropped using a syringe for 3 s. Meanwhile, another self-cleaning test was conducted using a window glass (7 × 4 cm^2^) coated with PIFSS-10. The coated glass was tilted at an angle of 20°. The standard sand (0.5 g) was sprayed and then DI water (1.5 mL) was dropped using a syringe for 5 s.

An adhesion cross-cut test was performed for the PIFSS coatings on glass slides according to ISO 2409. A right-angle lattice pattern is cut into the coatings (Figure S1 in Supplementary Material), penetrating through to the substrate. Six cuts in each direction of the coatings were made at a uniform cutting rate: the spacing of the cuts in each direction was 2 mm. The cut area of the samples was carefully examined visually.

The hardness of PIFSS-10 and PIFSS-15 coatings was determined by a pencil test according to ISO 15184. PIFSS coatings on glass slides (7.2 × 2.5 cm^2^) were prepared and placed on a horizontal surface. A pencil with a certain hardness was inserted in a test instrument, and the pencil was pushed in the direction away from the operator at a slow constant speed after the pencil has come to rest on the coating. The pencil lead was pushed over the paint surface at an angle of 45°, exerting a force of 7.35 ± 0.15 N on the surface. After cleaning all fragments of pencil lead from the coating surface, the operator assessed whether any damage occurred or not. The test result is the highest pencil hardness at which no marking occurs.

## 3. Results and Discussion

### 3.1. Preparation and Characterization of PIFSS Coatings

6FDA/TFMB polyimide is known to have high optical transparency, colorlessness and high glass transition temperature (>300 °C) [26]. 6FDA and TFMB were reacted in DMAc to give a polyamic acid solution and the polyamic acid was end-capped using 3TPA (Figure 1). Different amounts of a silica sol suspension was added to the end-capped polyamic acid solution to prepare PASS suspensions. The PASS suspensions were spin-coated on glass slides and thermally imidized to afford PISS coatings, which were then fluorinated using hydrolyzed TEFDS to give PIFSS coatings (Figure 2 and Table 1). The end-capping agent 3TPA was used to link silica sol particles to polyimide molecules to improve uniform distribution of silica sol particles in the polyimide matrix.

**Figure 3:**
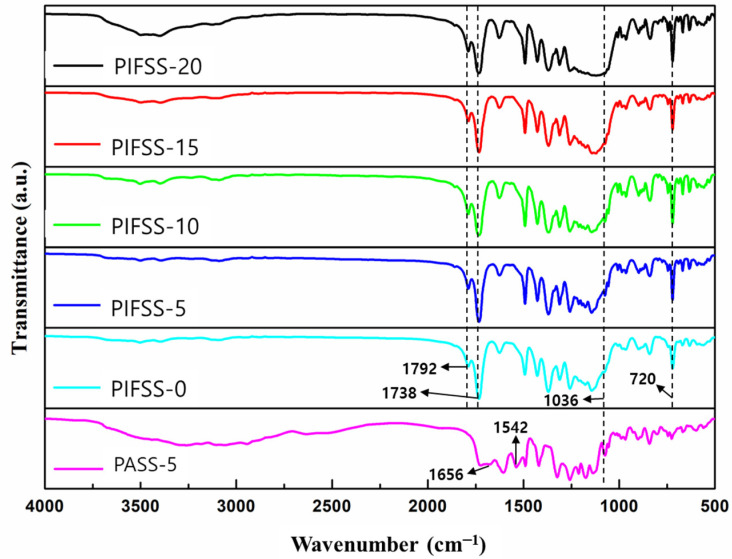
FT-IR spectra of PIFSS and PASS-5 coating samples.

The chemical structures of PASS-5 and PIFSS samples were confirmed by FT-IR spectroscopy in the range of 4000–500 cm^−1^ (Figure 3). The FT-IR spectrum of PASS-5 showed peaks corresponding to C=O stretching (1656 cm^−^^1^) and C–NH stretching (1542 cm^−^^1^), implying the formation of polyamic acid. All the PIFSS samples showed peaks corresponding to C=O asymmetric stretching (1792 cm^−1^), C=O symmetric stretching (1738 cm^−1^), and cyclic C=O bending (720 cm^−1^), indicating the formation of polyimide [30,31]. In addition, the Si-O stretching peaks at 1036 cm^−1^ were observed for both PASS-5 and PIFSS samples. The 1036 cm^−1^ peaks became stronger and broader as the silica sol content of the PIFSS samples was increased. These combined results indicate that the PIFSS samples were successfully prepared from the PASS samples. The FT-IR spectra of PIFSS samples were practically the same as corresponding PISS samples.

The resistance of PIFSS coatings to separation from glass substrate was evaluated by a adhesion cross-cut test according to ISO 2409 (Figure S1 in Supplementary Material). A right-angle lattice pattern is cut into the coatings and the spacing of the cuts in each direction was 2 mm. It was observed that the edges of the cuts were completely smooth and none of the squares of the lattice was detached. The excellent resistance of PIFSS coatings to separation is probably due to effective interaction such as hydrogen bonding between glass surface and the coatings. Meanwhile, all the PIFSS samples coated on the glass substrate were well soluble in polar aprotic solvents such as acetone, DMSO and DMAc. The coatings could be cleanly removed by simply dipping them in those solvents. It was demonstrated that repeatable coating of PIFSS could be achieved on the glass substrate from which a previous PIFSS coating was removed. This indicates that the glass substrate can be reused. In addition, the matrix 6FDA/TFMB polyimide showed high thermal stability up to about 500° (Figure S2 in Supplementary Material). Pencil hardness of PIFSS-10 and PIFSS-15 was measured according to ISO 15184, and they showed HB (average hard).

### 3.2. Self-Cleaning and Hydrophobic Properties of PIFSS Coatings

When water droplets contact a dirt-contaminated superhydrophobic surface, the dirt particles are carried away from the surface by rolling water droplets. It means the superhydrophobic surface has self-cleaning property. The superhydrophobicity can be achieved by the combination of high surface roughness and low surface energy. In this study, to increase the surface roughness and to decrease surface energy, silica sol particles and TEFDS were employed, respectively. However, as the surface roughness is increased, the optical transparency can be reduced. Therefore, the composition of PIFSS suitable for the best self-cleaning performance along with high transparency was investigated.

**Figure 4:**
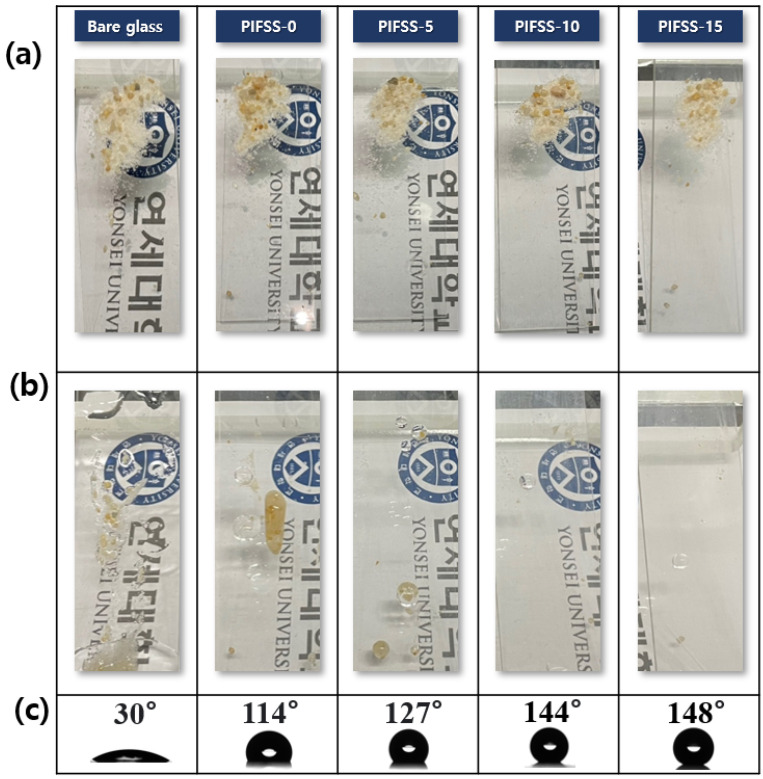
Self-cleaning and water contact angle properties of PIFSS coatings: (**a**) the photos of PIFSS coatings on which a standard sand was sprayed; (**b**) the photos of PIFSS coatings after dropping DI water (0.7 mL) for 3 s; (**c**) the water contact angles of the coating surfaces.

To study the influence of silica sol content on hydrophobicity, self-cleaning ability and optical transparency of the PIFSS coatings, the amount of TEFDS was fixed and the silica sol content was varied. The self-cleaning and hydrophobic properties of PIFSS samples were shown in Figure 4. PIFSS-20 could not be evaluated because of its brittleness. A bare glass showed a contact angle of 30°. The water contact angles of PIFSS-0, PIFSS-5, PIFSS-10 and PIFSS-15 were measured to be 114°, 127°, 144° and 148°, respectively. This means that as the amount of silica sol particles in the PIFSS coating was increased, the surface hydrophobicity increased. In particular, the surfaces of PIFSS-10 and PIFSS-15 had near superhydrophobic contact angles.

To investigate the self-cleaning property, the coating samples were tilted at an angle of 20°. A standard sand specified in ISO 679 (0.2 g) was sprayed on them as shown in Figure 4a, and then 0.7 mL of DI water was dropped using a syringe for 3 s. As shown in Figure 4b, the sands on bare glass, PIFSS-0 and PIFSS-5 were trapped in water droplets without flowing. Although the surfaces of PIFSS-0 and PIFSS-5 had hydrophobic contact angles, they showed insufficient self-cleaning properties at a tilt angle of 20° and under the spraying conditions (0.7-mL water, 3 s). In contrast, PIFSS-10 and PIFSS-15 coatings had good self-cleaning properties: the water droplets with sand pollutants rolled down the coating surfaces. These results showed that the silica sol contents of PIFSS-10 and PIFSS-15 were appropriate to have self-cleaning and high hydrophobic properties. To demonstrate possible application of PIFSS coating, a preliminary test was conducted using a window glass (7 × 4 cm^2^) coated with PIFSS-10 at a tilt angle of 20°. The standard sand (0.5 g) was sprayed and then DI water (1.5 mL) was dropped using a syringe for 5 s. It was observed that the water droplets with sand pollutants rolled down the coating surface, and the coated window glass was cleaned.

### 3.3. Transeparency of PIFSS Coatings

**Figure 5:**
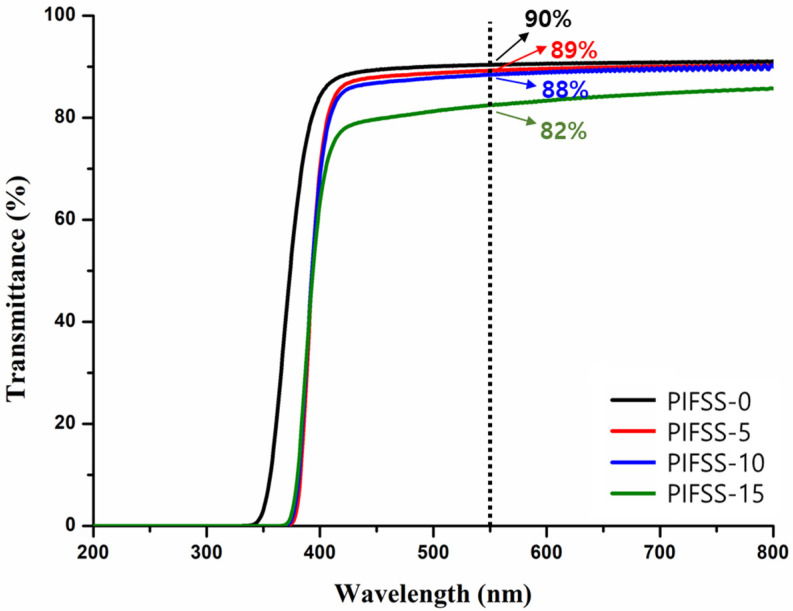
UV-Vis spectra of PIFSS coatings (thickness = about 1 μm) and the transmittance values at 550 nm: 90% (PIFSS-0); 89% (PIFSS-5); 88% (PIFSS-10); 82% (PIFSS-15).

Figure 5 shows the optical transmittance of PIFSS coatings in the range of 200~800 nm. At 550 nm, PIFSS-0, PIFSS-5, PIFSS-10 and PIFSS-15 showed transmittance values of 90%, 89%, 88% and 82%, respectively. PIFSS-20 sample was excluded because it was translucent and brittle. As the silica sol content was increased in PIFSS samples, the optical transmittance reduced. The reason of decreasing optical transmittance is that the silica sol particles in PIFSS samples make rough surface on coating. Such a roughness is detrimental to transparency because of light scattering. PIFSS-0, PIFSS-5 and PIFSS-10 showed similar transparency, while PIFSS-15 had much lower transmittance value. Based on the combined results of self-cleaning test and UV-Vis spectroscopy, PIFSS-10 coating is suitable as transparent self-cleaning coating.

### 3.4. Surface Mophology and Roughness of PIFSS Coatings

**Figure 6:**
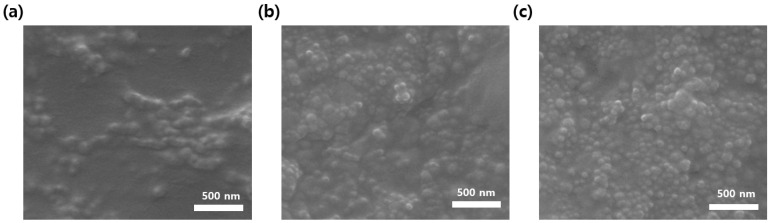
SEM images of coating surfaces. (**a**) PIFSS-5, (**b**) PIFSS-10 and (**c**) PIFSS-15.

In Section 3.2, it is described that PIFSS-10 and PIFSS-15 coatings showed good self-cleaning properties and nearly superhydrophobic contact angles (144° and 148°, respectively). In Section 3.3, the optical transmittance of PIFSS-15 was lower than that of PIFSS-10. These reasons were analyzed through SEM and AFM studies. The SEM images of the coating surfaces are shown in Figure 6. The image of PIFSS-0 is not shown because surface roughness was not observed and SEM study was not performed for PIFSS-20 coating because of brittleness. The SEM images (Figure 6a–c) showed that less than 200-nm particles were protruded out of the polyimide matrix surfaces. It was considered that the surface roughness formed from silica sol particles. The particle distribution of the PIFSS-5 surface was less uniform compared to the PIFSS-10 and PIFSS-15 surfaces. This means that the silica sol content of PIFSS-5 is not enough to form sufficient surface roughness. These results prove that the self-cleaning and hydrophobic properties of PIFSS-5 were poor as described in Section 3.2.

**Figure 7:**
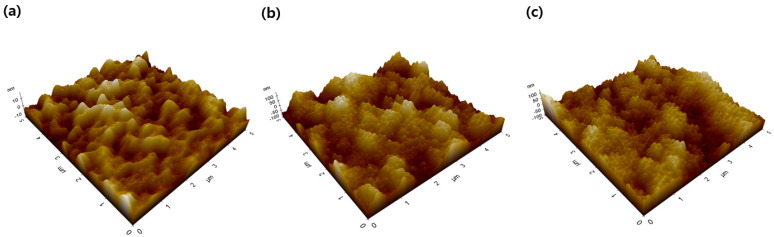
AFM 3D images of coating surfaces: (**a**) PIFSS-5, (**b**) PIFSS-10, and (**c**) PIFSS-15. The RMS roughness values of the coating surfaces were determined as (**a**) ~4.34 nm, (**b**) ~30.67 nm and (**c**) ~35.84 nm.

In Section 3.2 and Section 3.3, although the self-cleaning property of PIFSS-10 and PIFSS-15 was similar, PIFSS-15 showed much lower transparency than PIFSS-10. Since these properties are known to be related to surface roughness, 3D AFM study was conducted to investigate the surface roughness of the PIFSS coatings (Figure 7). The root-mean-square (RMS) roughness value of PIFSS-5 was 4.34 nm, which was considered too low to give self-cleaning property. In contrast, the RMS roughness values of PIFSS-10 and PIFSS-15 were much higher (30.67 nm and 35.84 nm, respectively), to afford good self-cleaning property. However, the higher RMS roughness value of PIFSS-15 leads to the lower transparency compared to PIFSS-10. It means that the surface roughness of PIFSS-10 is appropriate for high transparency and good self-cleaning property.

The nearly superhydrophobic property of PIFSS-10 is attributable to the surface roughness originated from micro-/nano-sized ridges and valleys on the coating surface as shown in Figure 7b. When a water droplet is placed on the coating surface, the micro-/nano-sized ridges and valleys lead to a discontinuation of the contact area between the water droplets and the coating surface as in the case of Cassie’s model [32,33,34]. The high water contact angle was thus achieved, and the adhesion force between the water droplet and the substrate was very small, resulting in good self-cleaning performance.

## 4. Conclusions

PASS suspensions were synthesized by adding different amounts of the silica sol suspension to each end-capped polyamic acid solution. Self-cleaning PIFSS coatings (PIFSS-0, PIFSS-5, PIFSS-10, PIFSS-15 and PIFSS-20) were prepared by coating of the PAAS suspensions, subsequent thermal imidization and TEFDS treatment. The thermal stability of the matrix polyimide (PIFSS-0) was excellent, and all of the PIFSS coatings showed very high resistance to separation from glass substrate. The coatings could be removed by simply dipping them in polar aprotic solvents, and repeatable coating of PIFSS could be achieved on the glass substrate, indicating that the glass substrate can be reused. The hydrophobic contact angle of PIFSS coating increased with increasing the amount of silica sol. Among them, PIFSS-10 and PIFSS-15 coatings showed nearly superhydrophobic contact angles (144° and 148°, respectively) and good self-cleaning property. It was confirmed by SEM and AFM studies that their hydrophobic and self-cleaning properties are due to uniform particle distribution and relatively high surface roughness (RMS roughness values are higher than 30 nm). However, PIFSS-15 showed much lower optical transparency due to excess silica sol content in the coating. Therefore, in this study, PIFSS-10 is the most suitable as transparent self-cleaning coating. The PIFSS nanocomposite coatings are expected to be applied in solar cell panels, windows, lenses and safety goggles. A disadvantage of our study is that the spin-coating was only attempted. To enlarge the application range of PIFSS coatings, the other coating methods such as spray coating will be tested and reported elsewhere.

## Figures and Tables

**Table 1:** Polyimide-fluorinated silica sol (PIFSS) coatings and their appearance.

Sample Code	Amount of Silica Sol Suspension (g) ^a^	Appearance ^b^
PIFSS-0	0	Transparent
PIFSS-5	5	Transparent
PIFSS-10	10	Transparent
PIFSS-15	15	Slightly opaque
PIFSS-20	20	Translucent and brittle

^a^ The amount of silica sol suspension added to 200 g of end-capped polyamic acid solution. ^b^ Investigated by naked-eye observation.

## Data Availability

The data presented in this study are available in this study. Additional information could be available on request from the corresponding author.

## Supplementary Materials

The following are available online at https://www.mdpi.com/article/10.3390/polym13234100/s1, Figure S1. Results of a cross-cut test for PIFSS coatings that was conducted according to ISO 2409: (a) PIFSS-0; (b) PIFSS-5; (c) PIFSS-10; (d) PIFSS-15. The spacing of the cuts in each direction was 2 mm.; Figure S2. TGA thermogram of a PIFSS-0 coating sample.
