# Supplementary material for: Transparent Self-Cleaning Coatings Based on Colorless Polyimide/Silica Sol Nanocomposite
Source: Polymers (Basel). 2021 Nov 25;13(23):4100. doi: 10.3390/polym13234100 (PMC8659165; doi:10.3390/polym13234100)
Supplement: Supplementary file 1 [file polymers-13-04100-s001.zip › polymers-1468677-supplementary.pdf]

Supplementary Material

Transparent Self-Cleaning Coatings based on Colorless Polyimide/Silica Sol Nanocomposite

Yun-Je Choi, Ju-Hee Ko, Seung-Won Jin, Hyun-Soo An, Dam-bi Kim, Kang-Hoon Yoon and Chan-Moon Chung *

Department of Chemistry, Yonsei University, Wonju, Gangwon-do 26493, Republic of Korea;

* Correspondence: cmchung@yonsei.ac.kr; Tel.: +82-033-760-2266

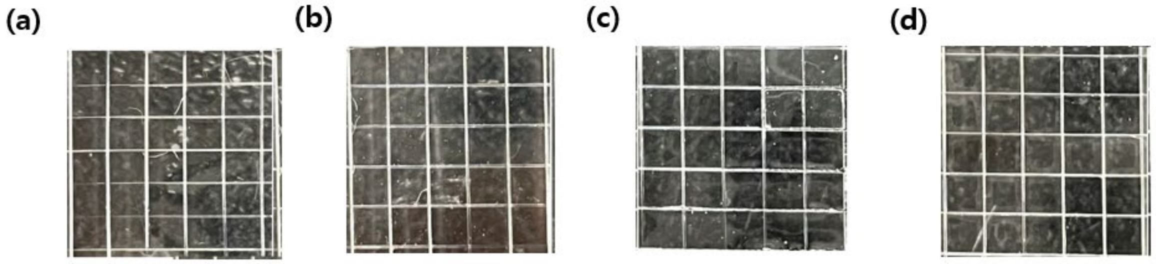

Figure S1. Results of a cross-cut test for PIFSS coatings that was conducted according to ISO 2409: (a) PIFSS-0; (b) PIFSS-5; (c) PIFSS-10; (d) PIFSS-15. The spacing of the cuts in each direction was 2 mm.

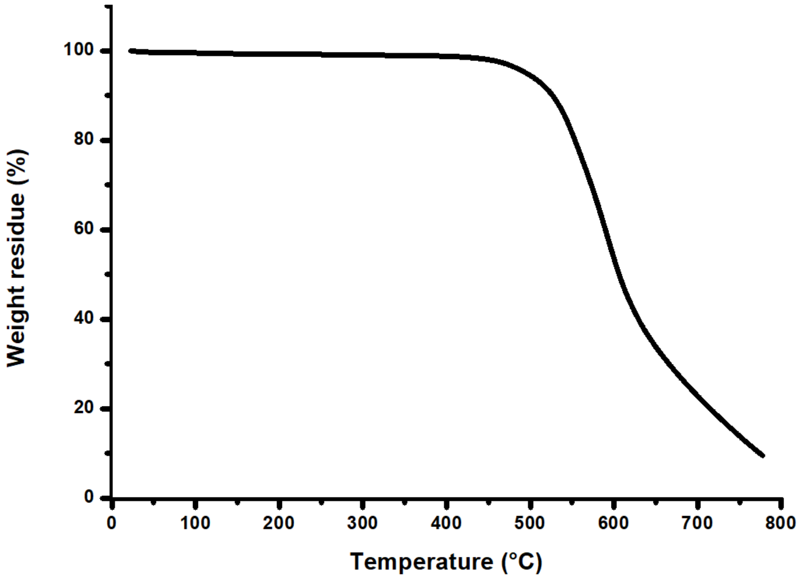

Figure S2. TGA thermogram of a PIFSS-0 coating sample.
